# The Best Part of Man’s Life

**Published:** 1881-10

**Authors:** T. F. Bayard


### THE BEST PART OF MAN'S LIFE.

It has been my lot for many years to assist in making laws for the government of this country, but the more I consider the problems of social and political arrangement and the forces that most influence and control it, the less do I find the statute books have to do in the regulation of the actual lives and occupations of the people. I mean how few of these occupations which engross the greater portion of our time, cause our labors and anxious considerations, in which we are most deeply interested, spend most of our money and bestow our powers in every way, are those to which any statute law or constitution compels us. The best part of man's life is in the world of his natural affections, and that realm has laws of its own that neither know nor heed king, kaiser, nor president, nor reichstags, nor congresses, and are deaf even to the voices of shouting popular majorities, but heed and obey rather the gentle voice of woman and the cry of helpless and feeble childhood.—*T. F. Bayard.*

